# Life-Threatening Lower GI Bleeding From Sigmoid Colon Metastasis of Cutaneous Angiosarcoma: A Case Report

**DOI:** 10.7759/cureus.105349

**Published:** 2026-03-16

**Authors:** Aqeel Roomy, Frans Pretorius

**Affiliations:** 1 General Surgery, Goulburn Valley Health, Shepparton, AUS

**Keywords:** angiosarcoma, colonic angiosarcoma, colonic metastasis, hartmann’s procedure, lower gastrointestinal bleeding, metastatic angiosarcoma, sigmoid colon metastasis, sigmoid colon tumor, transfusion-dependent anemia, vascular sarcoma

## Abstract

Angiosarcoma is a rare and aggressive malignancy of vascular endothelial origin, characterized by rapid progression and early metastatic dissemination. GI involvement is uncommon, and colonic metastasis from a cutaneous primary is exceptionally rare. We report the case of an 85-year-old man with a history of cutaneous angiosarcoma of the right cheek who presented with recurrent rectal bleeding and transfusion-dependent anemia. Colonoscopy demonstrated a large, ulcerated lesion in the sigmoid colon, causing luminal narrowing and active hemorrhage. Preoperative fluorodeoxyglucose PET-CT demonstrated focal thickening of the sigmoid colon with mild metabolic uptake, as well as increased uptake within the left pleura, raising suspicion for metastatic disease. Histopathological examination of the sigmoid lesion confirmed metastatic angiosarcoma. In the setting of ongoing bleeding and hemodynamic compromise, the patient underwent an urgent Hartmann’s procedure, which achieved definitive hemorrhage control. The postoperative course was uncomplicated, with significant clinical improvement. Despite the typically aggressive behavior associated with angiosarcoma, the patient remains clinically well nearly two years following resection. This case highlights the rare occurrence of colonic metastasis from cutaneous angiosarcoma presenting as life-threatening lower GI bleeding and underscores the role of surgical resection in achieving effective hemorrhage control and meaningful clinical outcomes in selected patients.

## Introduction

Angiosarcoma is a rare, high-grade malignant neoplasm arising from vascular endothelial cells, accounting for approximately 1-2% of all soft tissue sarcomas [1,2]. Cutaneous angiosarcoma predominantly affects elderly patients and most commonly involves the head and neck region [3]. The disease is characterized by aggressive local behavior and a propensity for early metastasis. GI involvement by angiosarcoma is rare, and colonic metastasis is exceptionally uncommon, with only a limited number of cases reported in the literature. When present, GI angiosarcoma often manifests with nonspecific symptoms, including anemia, abdominal pain, or overt GI bleeding, frequently resulting in delayed diagnosis. We report a rare case of metastatic cutaneous angiosarcoma involving the sigmoid colon presenting with life-threatening lower GI bleeding and discuss the diagnostic and management challenges associated with this uncommon presentation.

## Case presentation

An 85-year-old man with multiple comorbidities presented with recurrent episodes of rectal bleeding associated with symptomatic anemia requiring multiple blood transfusions. His medical history was significant for cutaneous angiosarcoma of the right cheek, previously treated with local excision followed by adjuvant radiotherapy at a tertiary oncology center. The patient remained under ongoing surveillance with medical oncology.

**Figure 1:**
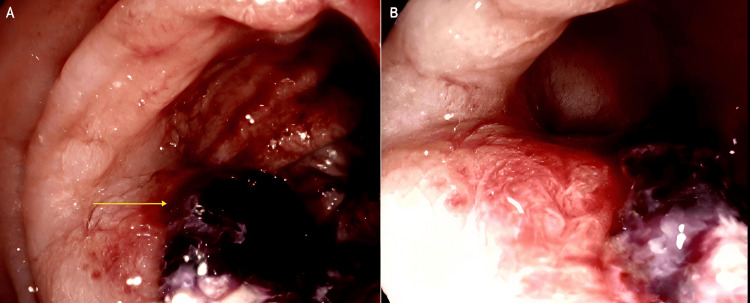
Colonoscopic appearance of a bleeding sigmoid lesion (A) Colonoscopic image demonstrating a large ulcerated and friable lesion within the sigmoid colon, approximately 30 cm from the anal verge. The arrow indicates the hemorrhagic tumor surface with adherent clot and mucosal disruption. (B) Colonoscopic view demonstrating the exophytic component of the tumor, causing significant luminal narrowing within the sigmoid colon, which prevented advancement of the colonoscope.

**Figure 2:**
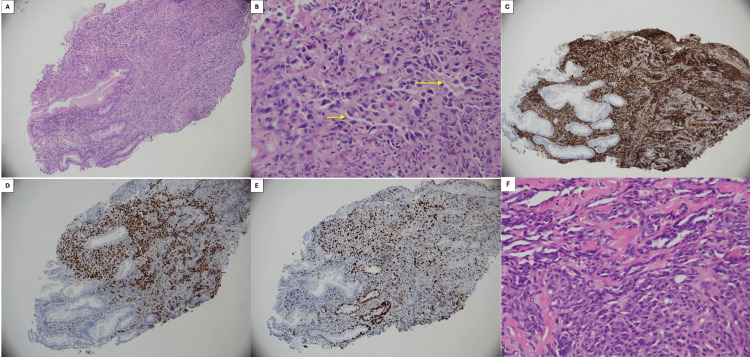
Histopathological features of metastatic angiosarcoma involving the sigmoid colon (A) Low-power H&E staining of the sigmoid colon biopsy specimen (100× magnification) demonstrating diffuse infiltration of the colonic mucosa by atypical malignant cells. (B) High-power H&E image (400× magnification) demonstrating markedly pleomorphic tumor cells with epithelioid to spindle morphology. The tumor cells form irregular and anastomosing vascular channels (arrows) lined by atypical endothelial cells with focal hobnail morphology, features characteristic of angiosarcoma. (C) Immunohistochemical staining for CD31 demonstrating strong membranous positivity within tumor cells, confirming endothelial differentiation. (D) Immunohistochemical staining for ERG demonstrating diffuse nuclear positivity within tumor cells, further supporting vascular endothelial origin. (E) Ki-67 immunostaining demonstrating a high proliferative index (>50%) within tumor nuclei. (F) Histological section from the patient’s previously resected primary cutaneous angiosarcoma (H&E stain, 400× magnification) demonstrating pleomorphic spindle-to-epithelioid tumor cells with focal areas of vasoformation. The morphological features are consistent with those observed in the sigmoid colon biopsy, supporting the diagnosis of metastatic angiosarcoma.

**Figure 3:**
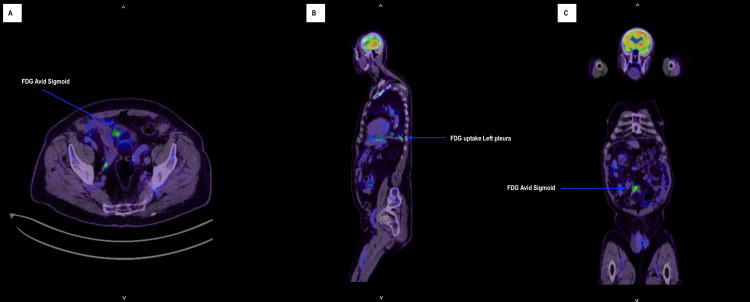
Preoperative FDG PET-CT demonstrating a metabolically active sigmoid lesion and pleural uptake (A) Axial fused FDG PET-CT image demonstrating focal increased FDG uptake within the sigmoid colon corresponding to the clinically identified lesion (arrow). (B) Sagittal fused FDG PET-CT image demonstrating increased metabolic uptake within the left pleura corresponding to areas of pleural thickening (arrow), raising suspicion for metastatic disease. (C) Coronal fused FDG PET-CT image demonstrating focal FDG uptake within the sigmoid colon consistent with the metabolically active sigmoid lesion (arrow). FDG, fluorodeoxyglucose

In early 2024, the patient underwent a colonoscopy for persistent rectal bleeding and altered bowel habits. This demonstrated a large ulcerated and friable lesion within the sigmoid colon, approximately 30 cm from the anal verge, associated with venous oozing and significant luminal narrowing that prevented traversal of the colonoscope (Figure 1). Histopathological examination of the biopsy specimen demonstrated a poorly differentiated malignant vascular tumor composed of pleomorphic epithelioid and spindle cells forming irregular vascular channels. Immunohistochemistry showed strong membranous CD31 positivity and diffuse nuclear ERG staining with a high proliferative index (Ki-67 >50%). In the context of the patient’s prior cutaneous angiosarcoma, these findings were consistent with metastatic angiosarcoma (Figure 2). Preoperative fluorodeoxyglucose (FDG) PET-CT demonstrated focal thickening of the sigmoid colon with mild metabolic uptake corresponding to the known lesion. Increased FDG uptake was also noted within the left pleura corresponding to areas of partially calcified pleural thickening, raising suspicion for metastatic disease (Figure 3). CT angiography did not clearly delineate the sigmoid lesion due to unprepared bowel, and no arterial contrast blush or delayed contrast extravasation was identified to suggest active bleeding at the time of imaging.

**Figure 4:**
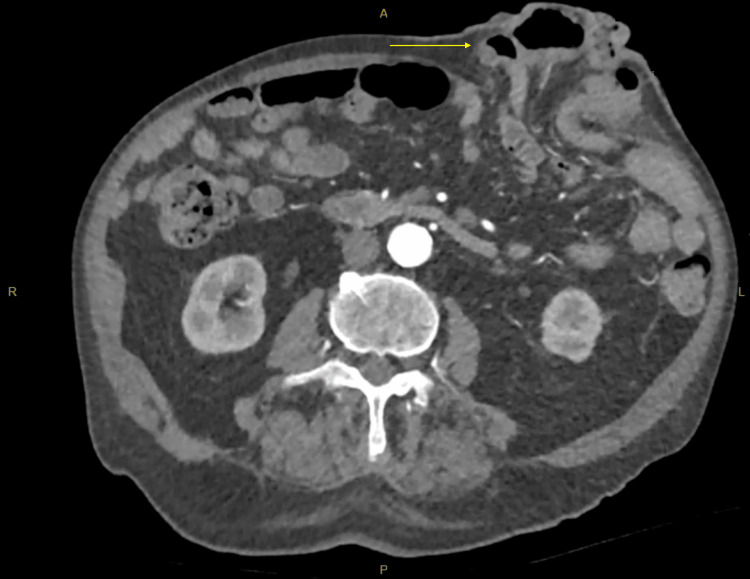
Parastomal hernia following Hartmann’s procedure CT of the abdomen and pelvis image demonstrating a parastomal hernia (arrow) adjacent to the end colostomy following Hartmann’s procedure. The hernia is characterized by a protrusion of abdominal contents through the fascial defect surrounding the stoma site, resulting in a visible bulge of the abdominal wall.

The patient subsequently developed worsening rectal bleeding with a hemoglobin level of 67 g/L, requiring blood transfusion. Given the transfusion-dependent bleeding and obstructing nature of the lesion, operative management was undertaken. The patient underwent a Hartmann’s procedure. Intraoperatively, the tumor was localized to the mid-sigmoid colon, and no macroscopic peritoneal metastases were identified. The postoperative course was uncomplicated, with resolution of rectal bleeding and significant clinical improvement. At follow-up in January 2026, the patient remained clinically stable with no recurrence of GI bleeding. A parastomal hernia was noted at the colostomy site and managed conservatively (Figure 4).

## Discussion

Angiosarcoma is an uncommon and highly aggressive malignant tumor arising from endothelial cells of vascular or lymphatic origin. It represents approximately 1-2% of all soft tissue sarcomas and most frequently involves the skin and superficial soft tissues, particularly in the head and neck region of elderly individuals [4]. These tumors exhibit aggressive biological behavior, characterized by rapid local invasion and a marked tendency for early hematogenous dissemination [5]. Consequently, clinical outcomes remain poor, with reported five-year survival rates ranging between approximately 35% and 45% [6].

Involvement of the GI tract by angiosarcoma is rare, and colorectal manifestations represent an exceptionally small proportion of reported cases. Since the first description of colonic angiosarcoma in the mid-20th century, only a limited number of cases have been documented [7]. Clinical presentation is often nonspecific and may include abdominal pain, anemia, or overt GI bleeding. Because these symptoms closely resemble those of more prevalent colorectal pathologies such as adenocarcinoma or inflammatory conditions, diagnosis is frequently delayed. In some cases, patients present with acute complications, including bowel obstruction or severe hemorrhage.

Definitive diagnosis relies on histopathological evaluation. Angiosarcomas display considerable morphological variability, with patterns ranging from irregular vascular channels lined by atypical endothelial cells to solid sheets of pleomorphic spindle or epithelioid cells. Poorly differentiated tumors may closely resemble other malignant entities, making diagnosis challenging on small biopsy specimens. Immunohistochemical staining, therefore, plays a crucial role in confirming endothelial differentiation [8]. Markers such as CD31, CD34, and ERG are commonly expressed and assist in distinguishing angiosarcoma from other poorly differentiated neoplasms. In our case, the tumor demonstrated strong CD31 and ERG expression together with a high proliferative index, findings consistent with metastatic angiosarcoma in the context of the patient’s previously diagnosed cutaneous primary [9].

Management strategies for colorectal angiosarcoma are not well defined due to the rarity of the disease. Surgical resection remains the cornerstone of treatment for localized disease and is frequently undertaken to control complications [10]. Achieving clear surgical margins can be challenging given the infiltrative growth pattern and potential multifocality of these tumors. The role of systemic therapy remains uncertain, although chemotherapeutic agents have demonstrated activity in advanced disease [11]. Radiotherapy may also be considered in selected cases; however, evidence supporting its survival benefit is limited [12]. Recent case reports and small series continue to emphasize the rarity and aggressive clinical course of GI angiosarcoma, with most evidence regarding diagnosis and management derived from isolated contemporary reports rather than large prospective studies [13]. This case adds to the limited literature describing GI involvement of cutaneous angiosarcoma and illustrates how metastatic disease may present with life-threatening lower GI hemorrhage. By highlighting this rare presentation, the report reinforces the importance of maintaining diagnostic vigilance in patients with a history of angiosarcoma who present with unexplained GI bleeding.

Metastatic involvement of the colon from cutaneous angiosarcoma is extremely rare and should be considered in patients with a history of angiosarcoma who present with unexplained GI hemorrhage. The variable histological appearance of angiosarcoma highlights the importance of immunohistochemical analysis in establishing the diagnosis. Surgical intervention may be required for symptom control, particularly in cases complicated by persistent bleeding. Given the limited number of reported cases, additional case reports remain important to improve understanding of the presentation, diagnosis, and management of this rare malignancy.

## Conclusions

Colonic metastasis from cutaneous angiosarcoma is exceptionally rare and can present with life-threatening GI hemorrhage. This case emphasizes the importance of considering metastatic disease in patients with a history of angiosarcoma who develop unexplained GI bleeding. Accurate diagnosis relies on careful histopathological evaluation with immunohistochemical confirmation due to the variable morphological features of angiosarcoma. In selected patients, surgical resection may provide definitive hemorrhage control and meaningful clinical benefit. Notably, this case demonstrates that favorable outcomes with durable disease control may be achieved despite the typically aggressive behavior of angiosarcoma.
